# Research Applying Adaptive Leadership Framework for Chronic Illness Across Settings of Care

**DOI:** 10.1093/geroni/igab046.647

**Published:** 2021-12-17

**Authors:** Ashley Leak Bryant, Anna Beeber, Bei Wu

## Abstract

The carepartner is an essential member of the team to facilitate and assist in maximizing the independence of the older adult. The four papers in this symposium applies The Adaptive Leadership Framework for Chronic Illness (ALFCI) at the point of design, as well as an analytic framework for literature synthesis, intervention design, and analysis of existing data. In the first paper, a qualitative metasummary of a scoping review synthesizes qualitative findings about fatigue adaptation and challenges for stroke survivors, care partners, and healthcare professionals. The second paper describes the use of the ALFCI in an intervention study to manage symptom challenges in older adults with acute myeloid leukemia. The third paper shares staff’s experiences of providing direct care for older residents with advanced dementia in long-term care facilities. The fourth paper describes use of the carepartner–assisted intervention to improve oral hygiene of older adults with cognitive impairment. The ALFCI is a useful framework for intervention design 1) this framework provides a comprehensive way to examine the context of symptoms/behaviors (not just the symptom/behavior in isolation), 2) the framework guides “collaborative work”, 3) analytically it can help guide development of shared meaning of communication and “collaborative work” of dyads (family caregivers, long-term care staff and older adults), and 4) helps understand process of staff utilizing their strengths and doing adaptive work to facilitate interactions and communication, managing older residents’ behavioral and psychological symptoms of dementia, and improving their care provision and work life.

